# Drug discovery research in Ghana, challenges, current efforts, and the way forward

**DOI:** 10.1371/journal.pntd.0010645

**Published:** 2022-09-15

**Authors:** Richard Kwamla Amewu, Patrick Amoateng, Patrick Kobina Arthur, Prince Asare, Isaac Asiamah, Daniel Boamah, Isaac Darko Otchere, Cedric Dzidzor Amengor, Edmund Ekuadzi, Kelly Chibale, Susan Jane Farrell, Regina Appiah-Oppong, Dorcas Osei-Safo, Kevin David Read, Ian Hugh Gilbert, Dorothy Yeboah-Manu

**Affiliations:** 1 Department of Chemistry, University of Ghana, Legon-Accra, Ghana; 2 Department of Pharmacology and Toxicology, School of Pharmacy, College of Health Sciences, University of Ghana, Legon-Accra, Ghana; 3 Department of Biochemistry, Cell and Molecular Biology, University of Ghana, Legon- Accra, Ghana; 4 Department of Bacteriology, Noguchi Memorial Institute for Medical Research, University of Ghana, Legon, Ghana; 5 Department of Chemistry, School of Physical Sciences, University of Cape Coast, Cape Coast, Ghana; 6 Department of Microbiology, Centre for Plant Medicine, Mampong-Akuapem, Ghana; 7 Department of Pharmaceutical Chemistry, School of Pharmacy, University of Health and Allied Sciences, Ho, Ghana; 8 Department of Pharmacognosy, Faculty of Pharmacy and Pharmaceutical Sciences, Kwame Nkrumah University of Science and Technology (KNUST), Kumasi, Ghana; 9 Holistic Drug Discovery and Development (H3D) Centre at the University of Cape Town (UCT), Rondebosch, South Africa; 10 Wellcome Centre for Anti-Infectives Research (WCAIR), Division of Biological Chemistry and Drug Discovery, University of Dundee, Dundee, United Kingdom; 11 Department of Clinical Pathology, Noguchi Memorial Institute for Medical Research, University of Ghana, Legon, Ghana; Gulu University, UGANDA

## Abstract

We have a long-term vision to develop drug discovery research capacity within Ghana, to tackle unmet medical needs in Ghana and the wider West African region. However, there are several issues and challenges that need to be overcome to enable this vision, including training, human resource, equipment, infrastructure, procurement, and logistics. We discuss these challenges from the context of Ghana in this review. An important development is the universities and research centres within Ghana working together to address some of these challenges. Therefore, while there is a long way to go to fully accomplish our vision, there are encouraging signs.

## Introduction

There is limited drug discovery research in sub-Saharan Africa [1], with the notable exception of Holistic Drug Discovery and Development (H3D), which is based in Cape Town. Hence, we recognized the value in outlining our vision and the progress made in Ghana towards the development of drug discovery research and to highlight some of the challenges that we face. There is a huge need for new drugs for Ghana and across sub-Saharan Africa, particularly in the anti-infective area. Diseases such as malaria, leishmaniasis, schistosomiasis, and tuberculosis (TB) have a massive impact in Ghana, and yet there is inadequate research in drug discovery into these diseases locally. Due to the economics of drug discovery research, there is little commercial gain to be made from drugs for such diseases. Furthermore, for noncommunicable diseases such as cancer, the approaches of diagnosis and management are often very different in countries such as Ghana, from high-income countries, necessitating research in these areas too.

Various efforts are underway in Ghana to increase drug discovery research efforts. In this article, we discuss the status quo and some of the challenges facing drug discovery research in Ghana. Furthermore, we discuss the opportunities going forward with emphasis on the preclinical development stages. Our overall vision is to grow the current infrastructure to enable integrated and professional drug discovery activities: screening of compounds, compound optimisation, and compound characterisation. The eventual aim is to be able to produce preclinical drug candidates.

**Fig 1:**
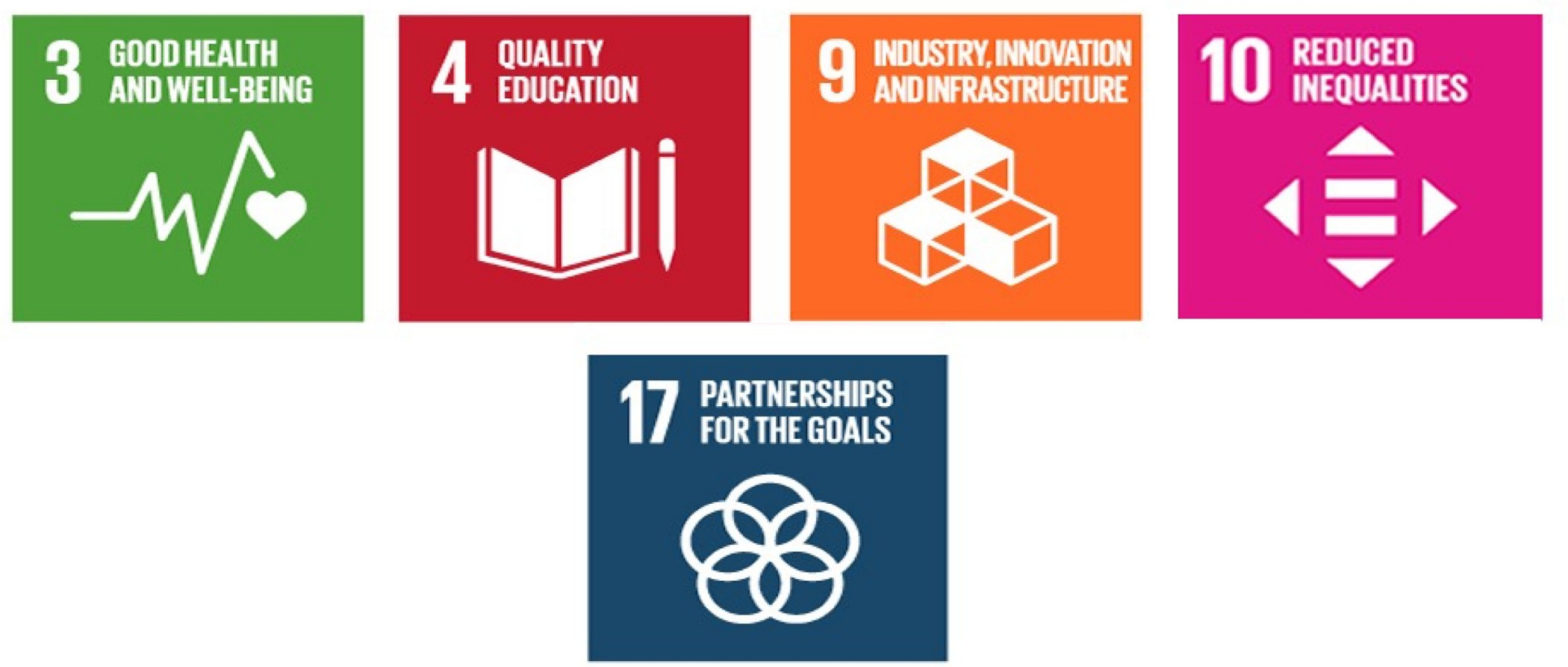
United Nations Strategic Development Goals, which can be impacted by Drug Discovery.

Developing a drug discovery research infrastructure will address some of the United Nations Strategic Development Goals (SDGs), highlighted in Fig 1 [2], giving many additional benefits to Ghana. These benefits include education and training across many disciplines to create an environment for Ghanaian researchers to flourish. Furthermore.it offers the potential for generation of “spin-out” companies research groups to generate innovation and economic development. Moreover, building up drug discovery research gives opportunities to strengthen South–South interactions within the West Africa subregion through collaborative research ultimately, we hope this work will lead to the discovery of candidate medicines.

**Fig 2:**
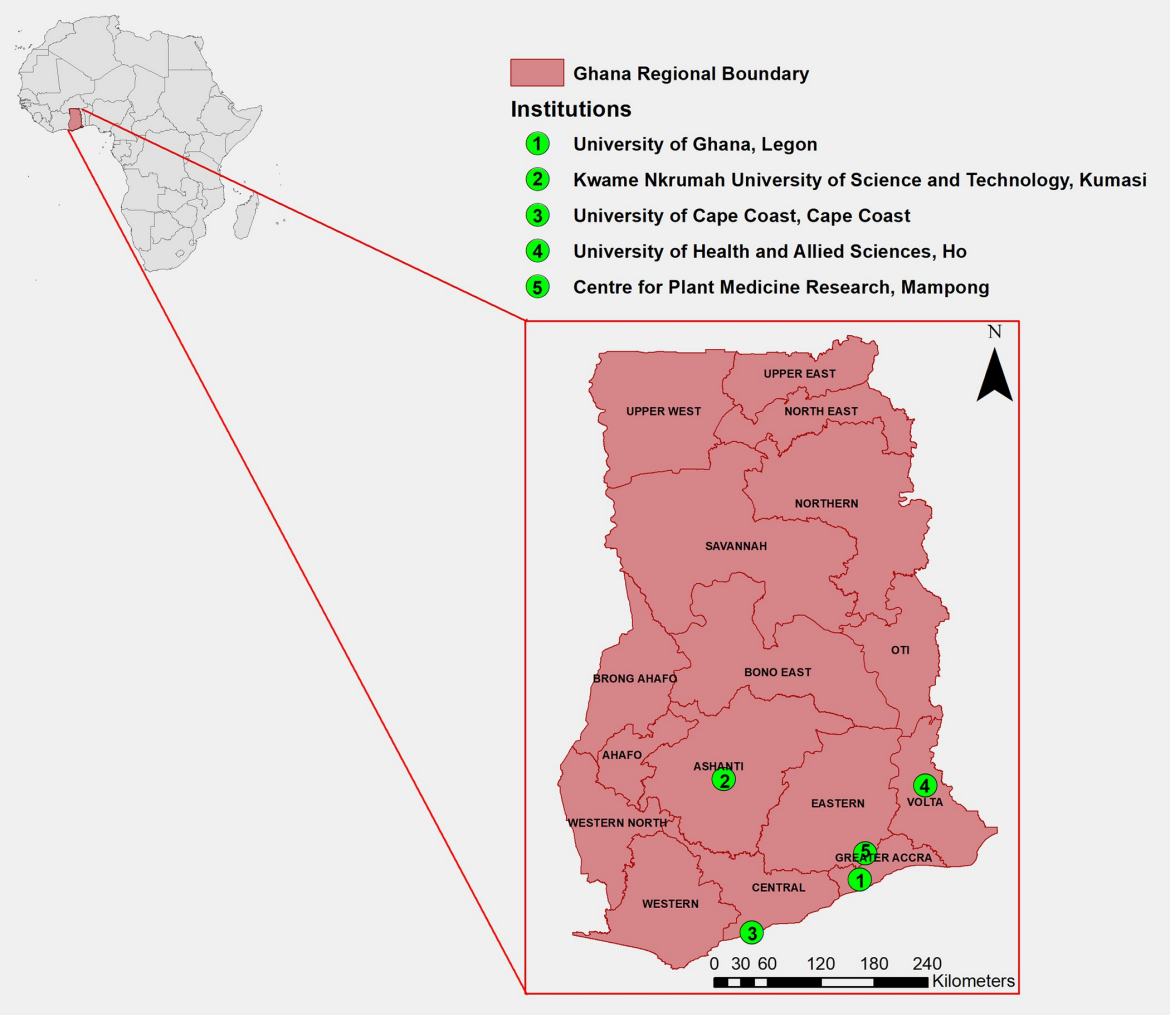
Universities and Institutes with an interest* in drug discovery in Ghana. *This was determined based on publications in drug discovery and through records of collaborations across the institutions. The base map used for constructing the map as well as the copyright information for use of the base map files are freely available at https://data.humdata.org/dataset/cod-ab-gha. No changes were made to the base map files.

In Ghana, there are a number of key universities and research institutions that have an ambition for drug discovery research (Fig 2). These include the University of Ghana (UG), Accra; Kwame Nkrumah University of Science and Technology (KNUST), Kumasi; University of Cape Coast (UCC), Cape Coast; and the University of Health and Allied Sciences (UHAS), Ho. A constituent part of UG is the Noguchi Memorial Institute of Medical Research (NMIMR), with excellent infrastructure, including Biosafety Level 3 (BSL3) laboratories, an animal experimentation facility for pathogen studies and clinical trials unit. In addition, the Centre for Plant Medicine Research (CPMR), Mampong carries out research on herbal medicines.

Drug discovery has multiple requirements, many of which have been initiated in Ghana. Pathogen screening has been developed at multiple sites. There is a significant amount of work, trying to take advantage of small molecules from natural sources such as plants, marine invertebrates, fungi, and bacteria. Some of this research is guided by traditional medicine, with the aim of identifying the active constituents. These chemical investigations have yielded many structurally unique compound classes including clerodane diterpenes [3], flavonoid glycosides [4], prenylated aryl benzofurans [5], indole alkaloids, [6] benzophenanthridines [7], dichapetalins [8–10], isoflavones [11], legonmycins [12], and accramycins [13,14] which have exhibited promising activities following screening in several anti-infective assays. More recently, drug discovery using small synthetic molecules is being developed. Furthermore, there are various research groups, elucidating the underlying mechanisms of diseases as well as identifying and validating targets for therapeutic interventions.

### Key challenges

There are several key challenges that we have identified as confronting drug discovery research in Ghana. We anticipate that many of these will be similar to other sub-Saharan African countries and that any knowledge gained should be shared to support research projects in the continent more widely.

### Training

Ghana has a well-developed and thriving tertiary education system, which trains people in the basic science disciplines such as chemistry, biochemistry, and microbiology. However, there is a need for training in more specialised aspects in areas such as drug discovery. Firstly, in how to carry out drug discovery research, understanding the different stages and requirements, and how to integrate the basic disciplines. Secondly, other required disciplines are largely absent, such as drug metabolism and pharmacokinetics (DMPK). People need to be trained in these techniques, and relevant assays need to be established in Ghana. It is also important people learn how to interpret and share these data with colleagues working in other disciplines and work together to make decisions on research project progression.

The younger generation, who are quicker to adopt modern technology, need to be trained to complement the efforts of the current scientists. Expertise in the use of robotics, data processing software, and computational chemistry techniques, including machine learning and artificial intelligence, are needed.

There is also a need for training in the use and maintenance of the scientific equipment. Some of the equipment in Ghana is underutilized, obsolete, or in poor working condition owing to an absence of expertise and regular maintenance. The underutilization of working equipment and frequent breakdowns of equipment point to the need to train more technical staff. Training needs to focus on hands-on use and maintenance to ensure maximum and proper utilization of equipment. Focussing the training on technical staff is advantageous as there is usually less turnover in this category of staff, although they manage and operate the equipment. Using bidirectional staff exchange programmes between industry and educational institutions’ technical staff can build the needed capacity at little cost.

### Human resource

There is little funding for researchers to carry out studies in Ghana. Most staff at the Principal Investigator level have high teaching and administrative duties, leaving little time for research. Funding for postdoctoral staff from internal university sources is limited, and external funding is often limited to larger, more competitive grant applications. PhD and Master’s students often have to self-fund due to a lack of funding opportunities, leading to many students not continuing in academia as they cannot afford to.

These funding issues lead to a culture of students carrying out placements or entire degrees at institutes outside of Ghana, especially in the drug discovery field since opportunities are limited. Some get the opportunity for further study in South Africa, but many move to Europe or the USA for further degrees. Many pharmaceutical companies and institutions run training schemes for limited periods, which is an excellent way to upskill individuals. However, without sufficient infrastructure and dedicated time for research at their home institution, it can be difficult to put learnings into practice.

All these factors culminate in limited number of individuals working in Ghana on drug discovery. The networking between universities is therefore essential to allow a critical mass of researchers across the country to work together to further drug discovery research.

### Equipment

Most of the critical equipment is in place, albeit not all are located at the same University (Table 1). However, there are still equipment needs, for example, LC-MS-MS equipment and robotic equipment for high-throughput compound screening. Some equipment needs to be duplicated between institutes, to prevent the delays and complexities of transferring samples between institutions.

**Table 1:** Key equipment in Ghana.

Institution	Equipment*
University of Ghana—Chemistry	500 MHz NMR LCMS, FTIR, Single Crystal X-Ray Diffractometer
University of Ghana—Noguchi Memorial Institute of Medical Research	Illumina MiSeg next-generation sequencer qRTPCR Machine, GridIon, Illumina Nextseq 2000 Flow cytometers Multicolour FACS Machine, Animal experimentation facility, MultiScan spectrophotometers, BSL-3 Laboratories, Atomic Absorption Spectrophotometer HPLC
Kwame Nkrumah University of Science and Technology	HPLC LCMS GCMS Refractometer FTIR UV-VIS 500 MHz NMR Flow Cytometer
University of Cape Coast—Chemistry	HPLC GCMS UV-VIS Flame Photometer FTIR Atomic Absorption Spectroscopy
Centre for Plant Medicine Research, CPMR	Freeze-dryer Biosafety cabinet (Class IIB)

*FTIR, Fourier transform infrared spectroscopy; GCMS, gas chromatography mass spectrometer; HPLC, high-performance liquid chromatography; LCMS, liquid chromatography mass spectrometer; NMR, nuclear magnetic resonance; qRTPCR, qualitative real-time polymerase chain reaction; UV-VIS, ultraviolet visible spectrometer.

Finding funding to purchase equipment can be problematic. While this is far from unique to Ghana, it is particularly problematic here, as individual universities cannot afford many pieces of equipment and it is very difficult to find external funding opportunities for large equipment grants. None of the major equipment suppliers have bases in West Africa, generally resulting in equipment being brought in, typically from Europe or the USA. When trying to decide on the procurement of large pieces of equipment, it becomes very challenging to demo equipment and ascertain its suitability. Once an item is purchased, installation engineers need to be flown in from abroad, which adds significantly to both cost and time. General servicing and maintenance can also involve flying in specialist engineers again adding to costs and often resulting in large periods where the instruments are not functioning. This whole process can be further hampered by the lack of availability of spare parts. Therefore, when purchasing any new equipment, funding should be identified to maintain them.

Going forward, it will be important to train engineers within Ghana to be able to service and maintain pieces of equipment. It would also be prudent to find a mechanism to supply the more commonly used spare parts within Ghana or at least within West Africa. Local support staff need to be trained in more sophisticated maintenance of the equipment. There also needs to be enhanced staff training in both the use of the equipment and interpretation of the data. Making this cross-institutional would facilitate a critical mass and allow institutions to aid one another. By way of example, at KNUST, a shared laboratory of equipment has been pooled together in the “KNUST Central Laboratory”. This facility allows researchers access to state-of-the-art technologies that would have been too expensive for individual departments to procure and maintain.

### Infrastructure

There are some promising signs in terms of infrastructure. The NMIMR has recently moved into a new state-of-the-art facility. The Central Laboratory at KNUST has very good modern laboratories, and there is a new University, the University of Health and Allied Sciences (UHAS), which has just built a new facility. The Science Annex building at UCC is near completion and is expected to provide modern laboratory space and facilities.

However, there is still a need for extra infrastructural development across all the Institutions in Ghana. For instance, there is a need for new chemistry laboratories for synthetic chemistry because the existing buildings are old and were not designed for synthetic chemistry research. In addition, the majority of the laboratory space in the existing building is dedicated to undergraduate teaching. The current chemistry research laboratories have limited space available for research activities. In the medium term, it would be possible to fit additional fume chambers into the existing laboratories to enable synthetic small molecule drug discovery research. However, in the long term, a new building with appropriate facilities for synthetic chemistry is required.

### Procurement

This is one of the major challenges facing scientists in Ghana. Quicker access to research items would ensure meeting project timelines and delivery of proposed project goals. However, the process of procurement in Ghana is complex, with many bureaucratic layers. Science research is data-driven and timebound; therefore, delays in getting consumables and supplies are a huge disadvantage for researchers in Ghana. It impacts heavily on making projections and planning future research activities.

Most research items cannot be purchased directly from within Ghana, and they must be imported. The local companies who sell most of these items are third-party agents who themselves must import into the country before selling to prospective end users. Importation is further plagued with additional layers of challenges including complex shipping arrangements. Sometimes shipping can be by air, but other times is required by sea, extending further the time for delivery. These delays and challenges can be problematic with temperature-sensitive goods where a cold chain is required. All these factors can sometimes lead to the actual cost of an item being significantly more expensive than the advertised price by the supplier or the manufacturer.

Another important challenge is the payment process. Often the institutions, and for that matter, the procurement law, demands that payments are made for goods that have been supplied, inspected, and certified by the requisite authorities [15]. However, the suppliers, particularly the foreign suppliers, demand payment in advance. These bottlenecks compel the researcher to go through a third party (those who subscribe to prefinancing) with the consequence of paying more for the same items. In limited instances where credit is granted, if the payment to the supplier takes longer than stated in the agreements, it can reduce the chance of the researcher benefiting from any future credit from that supplier.

### Logistics

There is willingness to collaborate across institutions, but there is a need to develop structures to enable this. Drug discovery research would involve management of facilities across institutions. For example, management of equipment scheduling; tracking of chemicals, reagents, and samples; and cost recovery for equipment use. To support this, we have compiled a list of all existing core equipment across institutions and their locations. Efforts are underway to develop scheduling management systems to allow researchers at different institutions to book time on core equipment at other institutions. We hope to use IT resources to proficiently handle booking on required equipment ahead of time. Chemical inventories could be integrated to allow researchers across institutions to easily see what is available and can be borrowed from other laboratories, but this would require discussions with management across institutions.

### Signs of promise and our vision

Our long-term vision is to develop drug discovery research capabilities in Ghana, to a level where we can produce preclinical candidates and move towards partnership for clinical studies.

A key requirement to realise this vision is for the research-active Universities and research centres to work together to address the challenges highlighted above. While this initiative has been hindered by the Coronavirus Disease 2019 (COVID-19) pandemic, we have now formed working groups to bring together key staff within the different institutions. These are synthetic chemistry, analytical chemistry, pathogen screening, and natural product chemistry. Further working groups are being considered, including DMPK and computational chemistry. In addition, we have put in place a cross-institutional working group, to look at more strategic issues. These working groups have several aims:

- to identify the resources that currently exist within the universities and make sure that this information is shared between the different universities.
- to share learnings among the institutions.
- to identify training requirements and to try and identify how these can be provided.
- to identify opportunities for interaction between the universities, and for joint projects.

By way of examples

- A need for training in the theory and use of liquid chromatography mass spectrometry (LCMS) was identified. An online training programme was developed and run specifically for the users of the instruments, by the WCAIR.
- Often all the equipment and expertise exist within Ghana, but not in the same institution. Thus, there is synthetic chemistry at UG, extensive analytical chemistry capabilities at KNUST, and pathogen screening at the Noguchi Institute, UG. Bringing these groups together provides a more powerful approach. For example, compounds synthesised in the Department of Chemistry UG were tested for antimicrobial activity at NMIMR [16]. In two separate collaborations, semi-synthesis of a natural product and an isolated plant-derived natural product, both carried out in the Department of Chemistry, UG, were assayed for antitrypanosomal activity at the West African Center for Cell Biology of Infectious Pathogens, UG [17,18]. In another study to access the efficacy some medicinal plants for the treatment of mycobacterial infections, stem back extracts isolated from KNUST were tested at NMIMR for antimicrobial activity and cytotoxicity [19].
- The University of Ghana is developing a database for their Natural Product work and are expanding this to include KNUST and then the other Universities and Institutions within Ghana.

Our vision is that by working together, we can start to overcome some of the challenges to develop further drug discovery research capabilities within Ghana and more widely within the West African region. Other challenges require infrastructural changes that are beyond our control. However, by coming together, we hope to speak in one voice and highlight these issues to all stakeholders for the desired change.

We are aiming to develop research programmes and to build our drug discovery research expertise and infrastructure around these programmes. Initially, this will be facilitated by interaction with external partners, such as the WCAIR and H3D Centre at the University of Cape Town (UCT) in South Africa, and support of funding agencies such as Medicines for Malaria Venture (MMV). However, as we develop our infrastructure and capabilities, we aim to internalise more of this research within Ghana. An excellent example of this approach is the way that H3D has developed at the UCT in South Africa. They initiated projects with MMV in malaria and developed a preclinical candidate. This was done in collaboration with others, such as the Swiss Tropical and Public Health Institute (SwissTPH). However, they have leveraged their success to eventually develop sophisticated malaria testing capabilities, DMPK, and clinical development experience. Another important aspect is to develop further our South–South interactions, particularly with researchers in the West African region.

We also think that it is important to focus on areas of unmet medical need that are relevant to Ghana and offer solutions that are appropriate to the context within Ghana. There are many infectious diseases that are poorly treated clinically and are of public health importance in Ghana. For example, Ghana is one of the countries with the highest levels of TB endemic countries in the world with an estimated national TB prevalence of 290 cases per 100,000 population and also a low case detection rate, estimated to be 20.7% [20]. This means that approximately 80% of TB cases go undiagnosed with its concomitant public health implications. Also, Ghana is among the 15 highest countries for malaria endemicity, with 2% of the global malarial cases and 3% deaths [21].

We have obtained funding from the Academy of Medical Sciences to allow networking within Ghana and to bring together senior representatives of our universities to support our vision. Unfortunately, like many things, this has been affected by the COVID-19 crisis. However, our initial meeting has led to the formation of online focus groups, and we hope to bring together people in person soon.

While developing drug discovery research in Ghana will undoubtably be a long road, we are encouraged by the steps we have taken so far. As we progress, further challenges will be identified but working together as a group of institutions, we hope that we can overcome these to fulfil our vision.

## References

[1] Chibale K, Wicht KJ, Woodland JG. Medicinal Chemistry Out of Africa. J Med Chem. 2021;64:10513–10516. doi: 10.1021/acs.jmedchem.1c01183 34296862

[2] https://www.un.org/sustainabledevelopment/news/communications-material/

[3] Annan K, Ekuadzi E, Asare C, Sarpong K, Pistorius D, Oberer L, et al. Antiplasmodial constituents from the stem bark of Polyalthia longifolia var pendula. Phytochem Lett. 2015;11:28–31.

[4] Ekuadzi E, Dickson R, Fleischer T, Annan K, Pistorius D, Oberer L, et al. Flavonoid glycosides from the stem bark of Margaritaria discoidea demonstrate antibacterial and free radical scavenging activities. Phytother Res. 2014;28(5):784–787. doi: 10.1002/ptr.5053 23970448

[5] Kyekyeku JO, Kusari S, Adosraku RK, Zühlke S, Spiteller M. Prenylated 2-arylbenzofuran derivatives with potent antioxidant properties from Chlorophora regia (Moraceae). Fitoterapia. 2016;108:41–47. doi: 10.1016/j.fitote.2015.11.013 26592854

[6] Mireku EA, Mensah ML, Mensah AY. Prenylated indole alkaloids from the stem bark of Hexalobus monopetalus. Phytochem Lett. 1989;2016(16):108–114.

[7] Addae-Mensah I, Munenge R, Guantai AN Comparative examination of two Zanthoxylum benzophenanthridine alkaloids for cardiovascular effects in rabbits. Phytother Res. 3(5):165.

[8] Osei-Safo D, Dziwornu GA, Appiah-Opong R, Chama MA, Tuffour I, Waibel R, et al. Constituents of the roots of Dichapetalum pallidum and their anti-proliferative activity. Molecules. 2017;22(4):532/1. doi: 10.3390/molecules22040532 28346380 PMC6154325

[9] Osei-Safo D, Dziwornu GA, Salgado A, Sunassee SN, Chama MA. Bi- and bisbibenzyls from the roots of Dichapetalum heudelotii and their antiproliferative activities. Fitoterapia. 2017;122:95–100. doi: 10.1016/j.fitote.2017.09.001 28882670

[10] Osei-Safo D, Chama MA, Addae-Mensah I, Waibel R, Asomaning WA, Oppong IV. Dichapetalin M from Dichapetalum madagascariensis. Phytochem Lett. 2008;1(3):147150.

[11] Asomaning WA, Otoo E, Akoto O, Oppong IV, Addae-Mensah I, Waibel R, et al. Isoflavones and coumarins from Millettia thonningii. Phytochemistry. 1999;51(7):937–941.

[12] Huang S, Tabudravu J, Elsayed SS, Travert J, Peace D, Tong MH, et al. Discovery of a single monooxygenase that catalyzes carbamate formation and ring contraction in the biosynthesis of the legonmycins. Angewandte Chemie, International Edition. 2015;54(43):12697–12701. doi: 10.1002/anie.201502902 26206556

[13] Maglangit F, Fang Q, Leman V, Soldatou S, Ebe R, Kyeremeh K, et al. Accramycin A, a new aromatic polyketide, from the soil bacterium, Streptomyces sp. MA37. Molecules. 2019;24(18):p:3384. doi: 10.3390/molecules24183384 31533358 PMC6767120

[14] Maglangit F, Zhang Y, Kyeremeh K, Deng H. Discovery of new antibacterial accramycins from a genetic variant of the soil bacterium, Streptomyces sp. *MA37*. Biomolecules. 2020;10(10):1464. doi: 10.3390/biom10101464 33092156 PMC7590149

[15] Public-Procurement-Amendment-Act-2016-ACT663_RePrinted.pdf (ppa.gov.gh).

[16] Amewu RK, Ade CF, Okyere ID, Morgan P, Yeboah-Manu D. Synthesis and Initial Testing of Novel Antimalarial and Antitubercular Isonicotinohydrazides. Results in Chemistry. 2022;4:100257.

[17] Dofuor AK, Ademolue TS, Amisigo CM, Kyeremeh K, Gwira TM. Chemical Derivatization and Characterization of Novel Antitrypanosomals for African Trypanosomiasis. Molecules. 2021;26(15):4488. doi: 10.3390/molecules26154488 34361641 PMC8347361

[18] Twumasi EB, Akazue PI, Kyereme K, Gwira TM, Keiser J, Cho-NgwaI F, et al. Antischistosomal, antionchocercal and antitrypanosomal potentials of some Ghanaian traditional medicines and their constituents. PLoS Negl Trop Dis. 2020;14(12):e0008919. doi: 10.1371/journal.pntd.0008919 33382717 PMC7810346

[19] Amponsah IK, Atchoglo PK, Ackah RY, Fokou PVT, Aboagye SY, Yeboah-Manu D, et al. In vitro anti-Mycobacterium ulcerans and cytotoxic activities of some selected medicinal plants and an indoloquinoline alkaloid. Int J Mycobacteriol. 2021;10(1):60–65. doi: 10.4103/ijmy.ijmy_243_20 33707373

[20] World Health Organization. World Tuberculosis Report 2021. Geneva, World Hearlth Organization.

[21] World Health Organization. World Malaria Report 2021. Geneva: World Health Organization; 2021.

